# Anomalies in global network connectivity associated with early recovery from alcohol dependence: A network transcranial magnetic stimulation and electroencephalography study

**DOI:** 10.1111/adb.13146

**Published:** 2022-02-27

**Authors:** Jodie Naim‐Feil, Paul B. Fitzgerald, Mica Rubinson, Dan I. Lubman, Dianne M. Sheppard, John L. Bradshaw, Nava Levit‐Binnun, Elisha Moses

**Affiliations:** ^1^ Department of Physics of Complex Systems The Weizmann Institute of Science Rehovot Israel; ^2^ Sagol Center for Brain and Mind Baruch Ivcher School of Psychology, Interdisciplinary Center (IDC) Herzliya Israel; ^3^ Graeme Clark Institute and Department of Biomedical Engineering University of Melbourne Melbourne Victoria Australia; ^4^ Epworth Centre for Innovation in Mental Health Epworth Healthcare and Monash University Department of Psychiatry Camberwell Victoria Australia; ^5^ Turning Point, Eastern Health and Monash Addiction Research Centre, Eastern Health Clinical School Monash University Victoria Australia; ^6^ Monash University Accident Research Centre Monash University Clayton Victoria Australia; ^7^ Turner Institute for Brain and Mental Health, School of Psychological Sciences Monash, University Melbourne Victoria Australia

**Keywords:** alcohol dependence, brain stimulation, electroencephalography, global connectivity, long‐interval cortical inhibition, network analysis, transcranial magnetic stimulation

## Abstract

Although previous research in alcohol dependent populations identified alterations within local structures of the addiction ‘reward’ circuitry, there is limited research into global features of this network, especially in early recovery. Transcranial magnetic stimulation (TMS) is capable of non‐invasively perturbing the brain network while electroencephalography (EEG) measures the network response. The current study is the first to apply a TMS inhibitory paradigm while utilising network science (graph theory) to quantify network anomalies associated with alcohol dependence. Eleven individuals with alcohol‐dependence (ALD) in early recovery and 16 healthy controls (HC) were administered 75 single pulses and 75 paired‐pulses (inhibitory paradigm) to both the left and right prefrontal cortex (PFC). For each participant, Pearson cross‐correlation was applied to the EEG data and correlation matrices constructed. Global network measures (mean degree, clustering coefficient, local efficiency and global efficiency) were extracted for comparison between groups. Following administration of the inhibitory paired‐pulse TMS to the left PFC, the ALD group exhibited altered mean degree, clustering coefficient, local efficiency and global efficiency compared to HC. Decreases in local efficiency increased the prediction of being in the ALD group, while all network metrics (following paired‐pulse left TMS) were able to adequately discriminate between the groups. In the ALD group, reduced mean degree and global clustering was associated with increased severity of past alcohol use. Our study provides preliminary evidence of altered network topology in patients with alcohol dependence in early recovery. Network anomalies were predictive of high alcohol use and correlated with clinical features of alcohol dependence. Further research using this novel brain mapping technique may identify useful network biomarkers of alcohol dependence and recovery.

## Graphical abstract

The current study provides preliminary evidence of altered network connectivity in patients with alcohol dependence in early recovery. These network anomalies were predictive of high alcohol use and associated with clinical features of past alcohol use severity. Therefore, by combining brain stimulation techniques with network science, it may be possible to identify useful network biomarkers of alcohol dependence and recovery.

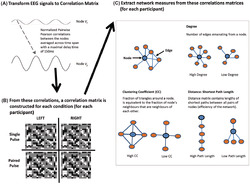

## 1 INTRODUCTION

Alcohol is a major contributor to disease burden and mortality in the global community.^1^ Alcohol dependence, a severe and chronically relapsing disorder, is characterised by a diminished capacity to inhibit alcohol consumption despite harms associated with continued use.^2^, ^3^, ^4^ Difficulty inhibiting the desire to drink typically persists beyond detoxification and is related to increased rates of relapse.^5^

Models of addiction have expanded the traditional neural mesolimbic ‘reward’ circuitry underlying addictive behaviours^6^ to also include two independent, yet interconnected brain systems: the limbic system in the incentive‐sensitisation of drugs^7^ and the prefrontal circuitry (PFC)^4^, ^8^, ^9^, ^10^ in regulating control over drug seeking behaviours. These cortical structures do not act in isolation, but rather, they recruit an interconnected network of brain regions. There is extensive research examining alterations within cortical structures of the mesocorticolimbic ‘reward’ circuitry;^6^, ^11^, ^12^, ^13^ however, research into the global features of this connectivity (i.e., how brain regions within the network communicate with each other) has been limited.

Network analysis (via graph theory) is a brain mapping approach which allows researchers to quantify network connectivity within the extensively interconnected human brain.^14^, ^15^, ^16^ By using network analysis techniques, it is possible to extract topological features of brain connectivity and examine network efficiency, integration and the strength of connections within a brain network.^14^, ^15^, ^17^ Recently, studies have begun to apply network analysis to functional magnetic resonance imaging (MRI) and diffusion‐tensor imaging (DTI) data to identify topological features of network disruption associated with alcohol consumption,^18^ dependence^19^ and abstinence.^20^, ^21^, ^22^ This approach has also been used to identify potential risk factors for alcohol dependence^23^, ^24^, ^25^ and examine how potential treatment approaches may impact these networks.^26^, ^27^ Across the majority of these studies, reductions in global efficiency,^18^, ^24^ local efficiency^20^, ^22^, ^25^, ^28^ and clustering coefficient,^20^, ^23^, ^25^ as well as the presence of abnormal functional hubs^29^ were observed in individuals with alcohol dependence relative to healthy controls. However, while these group differences were not found significant by all studies,^19^, ^22^, ^30^ a relationship between these network features and clinical aspects of alcohol dependence (such as alcohol use duration, severity of alcohol use and length of abstinence) has been identified.^19^

The studies described above construct network connectivity graphs from high spatial resolution neuroimaging data (acquired from an entire scan session). Electroencephalography (EEG), an economic and convenient neuroimaging tool with high temporal resolution, is capable of characterising brain network connectivity within a millisecond timescale.^31^, ^32^, ^33^ To date, only a limited number of EEG studies have explored the presence of altered network topology associated with short‐term^34^ and long‐term^35^, ^36^ alcohol consumption, and these studies have presented mixed findings. Therefore, preliminary studies, while providing broad support for the use of EEG to identify network anomalies, examined network connectivity from multiple levels (resting‐state and task‐activated) and utilised a diverse range of techniques (EEG and combined EEG‐MEG) and connectivity analyses (phase‐synchronisation index, coherence and cross‐correlation) as well as varied statistical analyses techniques (group‐wise comparisons and data mining models) which may account for discrepant results. In the current study, we propose that implementation of a more targeted approach, which directly activates the addiction circuitry while the network response is quantified, may provide further insight into the network topology associated with long‐term alcohol use.

Combined transcranial magnetic stimulation and electroencephalography (TMS‐EEG) is a novel technique which allows researchers to non‐invasively perturb the brain network (elicit a transient change in the network state) while EEG measures the network response within a millisecond timescale.^37^, ^38^, ^39^, ^40^, ^41^, ^42^, ^43^ Healthy controls typically present with a globally efficient and highly integrated network in response to TMS perturbations, while disturbances in network response have been identified across a range of psychiatric populations.^44^, ^45^, ^46^, ^47^, ^48^, ^49^ By utilising this novel approach, researchers are now capable of applying a more robust and direct perturbation to regions within the mesocorticolimbic ‘addiction’ circuitry. Previously, our research group delivered a paired‐pulse TMS paradigm (long interval cortical inhibition [LICI]^42^, ^50^, ^51^) to the frontal cortex of individuals with alcohol dependence (ALD) post‐detoxification to transiently inhibit cortical activity, while EEG measured the cortical response.^52^ This study provided the first direct report of altered cortical excitability (reduced cortical inhibitory [GABAergic] neurotransmission) localised within the frontal regions.^52^ However, as the frontal brain region does not act in isolation, it is possible that applying LICI to the frontal regions may also be capable of inducing a transient perturbation to the global network architecture. Therefore, the current study expands on this research and examines the *global effects* of the TMS‐perturbation on the distributed network activity of patients with ALD in early recovery.

Therefore, the current study had three major objectives: (i) Characterise global properties of network connectivity following a perturbation (TMS pulse) in patients with ALD in early recovery when compared to healthy controls, (ii) assess whether altered network features can predict the likelihood of membership in the ALD group and (iii) examine whether these global properties are related to clinical features of alcohol dependence.

## 2 MATERIALS AND METHODS

This collaborative research effort between Monash Alfred Psychiatry Research Centre, Turning Point and the Weizmann Institute of Science was approved by the Alfred Human Subjects Research and Ethics Committee. Participants were required to sign a detailed informed consent form prior to study enrolment and were informed that all participation was voluntary and they could withdraw at any time without prejudice.

### 2.1 Subjects

#### 2.1.1 Alcohol‐dependent sample

Eleven participants meeting criteria for DSM IV‐TR alcohol dependence (DSM‐IV‐TR, American Psychiatric Association 2000) within 2 years of successful completion of a detoxification programme (range = 8–668 days, median = 39 days) were recruited for the study. For initial screening, a phone call interview was conducted to assess psychiatric and medical history. Recruitment occurred through treatment agencies by self or clinician referral. Inclusion criteria were: (1) a *Wechsler Test of Adult Reading* standardised score higher than 100, to indicate no significant intellectual disability; (2) no drug or alcohol use (according to self‐report) since completion of the detoxification programme; and (3) no current comorbid mental health disorder (such as co‐morbid depression or psychosis). To confirm abstinence, the Timeline Follow Back (TFB) was administered, a 4‐week calendar where participants retrospectively reported their alcohol consumption over the previous month. Anyone reporting recent alcohol consumption via the TFB was excluded. Exclusion criteria also included acute medical or physical illness, engaging in pharmacotherapy treatment (including anti‐craving or anti‐depressant treatment), history of major depression or other drug dependence, reporting psychotic symptoms or suicidal ideation, head injury, epilepsy or history of seizures or metal implants.

#### 2.1.2 Healthy control sample

Sixteen healthy control subjects, without any previous or current history of alcohol/drug use disorder, psychiatric illness, head injury, epilepsy or seizures, were recruited through posters and local advertisements.

All participants received $40 reimbursement for their participation. Participants completed a general demographics questionnaire; the obsessive–compulsive drinking scale (OCDS; Anton et al., 1996)^53^ to assess levels of craving and the severity of alcohol dependence questionnaire (SADQ; Stockwell et al., 1979)^54^ for the level of dependence on alcohol; the Beck Depression Inventory (BDI; Beck and Steer 1987)^55^ to examine the presence of depressive symptoms^56^ and the Wechsler Test of Adult Reading (WTAR; Wechsler 2001)^57^ to assess intellectual functioning. Clinical and demographic information for both the ALD and control groups are reported in Table 1.

**TABLE 1:** Demographic and clinical data for all participants

	Healthy control (*n* = 16) mean ± SD	Alcohol dependent post‐detox (*n* = 11) mean ± SD	*p* value
Age	32 ± 6 years	40 ± 14 years	*p* = 0.06
Gender (male:female)	8:8	7:4	*p* = 0.484
AUD total	4 ± 4	11 ± 4	*p* < 0.01
SADQ	1 ± 1	28 ± 12	*p* < 0.01
BDI	2 ± 2	12 ± 12	*p* = 0.03
RMT	52.44 ± 7.82	49.91 ± 6.20	*p* = 0.380
AMT	44.31 ± 7.54	43.64 ± 6.68	*p* = 0.813
1 mV measure	61.75 ± 9.81	58.7 ± 6.86	*p* = 0.386
LICI % inhibition (left DLPFC)	27.72 ± 30.58	−23.78 ± 74.20	*p* = 0.008
LICI % inhibition (right DLPFC)	12.51 ± 40.69	−13.37 ± 77.23	*p* = 0.058

*Note*: AUD total = Alcohol Use Disorder Scale composite score; SADQ = Severity of Alcohol Dependence Questionnaire; BDI = Beck's Depression Inventory; RMT = Resting Motor Threshold; AMT = Active Motor Threshold; LICI = Long‐interval Cortical Inhibition (comparisons conducted while controlling for BDI). A LICI score greater than 0 reflects an inhibitory effect (with 100 being maximum inhibition) while a LICI score less than 0 presents a facilitatory effect.

### 2.2 Experimental design and techniques

For the current study, network analysis techniques were applied to TMS‐EEG data previously collected and published.^52^ A brief overview of these data collection processes is also detailed in the current publication.

#### 2.2.1 Transcranial magnetic stimulation

Biphasic TMS pulses were applied to the cortex via a figure‐of‐eight cooled coil connected to the MagPro R30 stimulator with a MagOption unit (Magventure, Denmark). The TMS coil was held over the scalp, with the handle of the coil pointed backwards, angled approximately 45° from the midsagittal line and held perpendicular to the presumed direction of the central sulcus. In a single session, active TMS was applied to the left motor cortex (to ascertain the appropriate stimulation parameters) and this was followed by stimulation of the right dorsolateral prefrontal cortex (PFC) and the left dorsolateral PFC. The order of the stimulation site (right before or after left dorsolateral PFC) was counterbalanced across participants.

#### 2.2.2 Motor cortical stimulation parameters

For motor cortical stimulations, TMS was applied to the left motor cortex while electromyography (EMG) recorded the motor‐evoked potentials (MEP) response via disposable disc electrodes placed over the contralateral first dorsal interosseous (FDI) muscle. EMG was recorded using Signal software (Cambridge Electronics Design, CED Micro 1401 mk II analogue‐to‐digital converting unit, Cambridge, UK), amplified and filtered (low pass 2 kHz, high pass 10 Hz) by a powerlab/4sp system (AD Instruments, Colorado Springs, CO) and processed offline. Single pulse stimulation was applied to the left motor cortex to determine the Resting Motor Threshold (RMT), Active Motor Threshold (AMT) and 1 mV peak‐to‐peak (measured over cap). RMT is determined by the minimum stimulus intensity required to elicit peak‐to‐peak MEP > 50 μV as measured by the contralateral FDI muscle in at least 5/10 trials (Rossini et al., 1994). AMT is determined as the minimum stimulation intensity, during active FDI muscle contraction (uniformly pressing index finger against a spring), needed to induce peak‐to‐peak MEPs of >100 μV in 3/5 consecutive trials. For the 1‐mV peak‐to‐peak measure, stimulator intensity is adjusted until it elicits peak‐to‐peak MEP of approximately 1 mV over 10 consecutive trials; this was conducted over the EEG cap (1 mV measure over cap) and measured by the contralateral FDI muscle contraction to determine the appropriate stimulation parameters for the application of frontal TMS‐EEG.

#### 2.2.3 Frontal stimulation measurement

TMS‐evoked cortical activity within the frontal regions was measured through a custom‐made 24‐channel EEG cap and recorded by the Synamps2 EEG system (Compumedics Neuroscan, TX, USA). All electrodes (sintered Ag/AgCl) were fixed in plastic electrode clips according to the standard 10–20 positions (EASYCAP GmbH, Germany) and were referenced to an electrode placed posterior to the Cz electrode (the connectivity analysis approach described below also utilised this standard referencing approach). All EEG signals were recorded DC at a sampling rate of 20 kHz and filtered through a low pass filter of 3500 Hz. Following TMS discharge, recharging capacitors were set to a 1‐s delay, to minimise TMS‐related artefacts in the EEG data. To reduce any potential TMS‐induced auditory effect, earphones with white noise (95 dB) were worn by all participants (Fitzgerald et al., 2008).

#### 2.2.4 Measurement of frontal cortical inhibition

**FIGURE 1:**
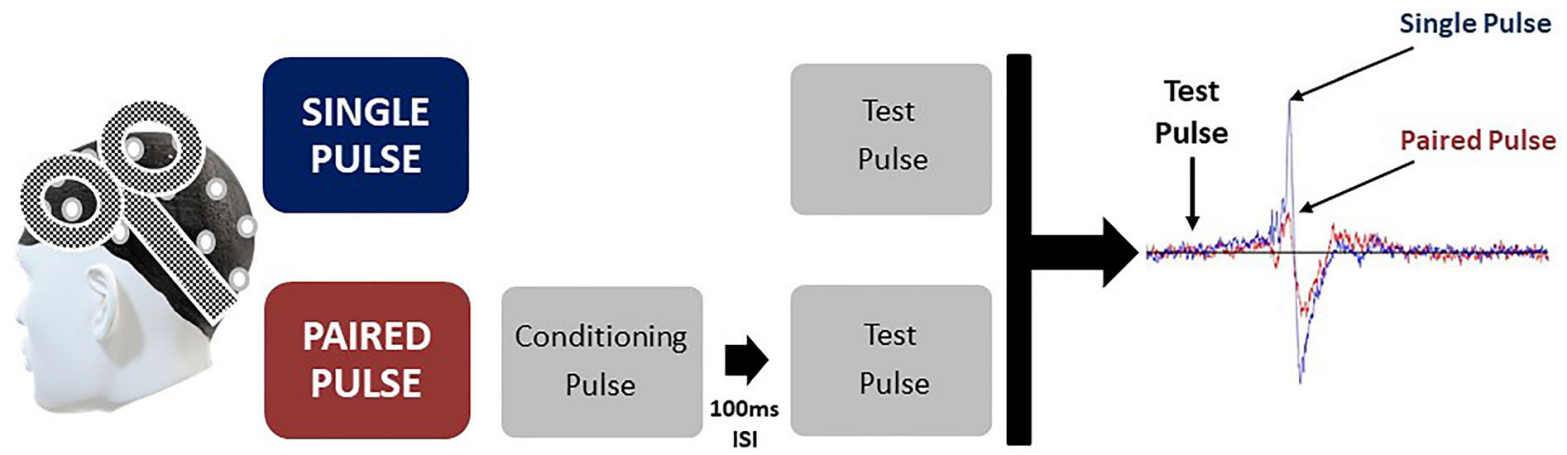
An illustration of the long interval cortical inhibition (LICI) paradigm. EEG recordings of cortical responses following: (A) Single pulse and (B) Paired‐pulse stimulation. The test pulse is preceded by the conditioning pulse, which suppresses cortical excitability. This illustration depicts the suppressed electroencephalography (EEG) response which is normally observed in control subjects following administration of LICI protocols with an inter‐stimulus interval (ISI) of 100 milliseconds

Long interval cortical inhibition (LICI) is an inhibitory paired‐pulse TMS paradigm which delivers two suprathreshold pulses with the stimulus intensity based on the 1‐mV peak‐to‐peak measure (established by the motor stimulations). The process involves applying the suprathreshold *conditioning* pulse, then the inter‐stimulus interval (ISI) of 100 ms, which is followed by a suprathreshold *test* pulse. This results in the conditioning pulse suppressing the cortical response produced by the test stimulus. This process is illustrated in Figure 1.

For this protocol, both single and paired‐pulse frontal stimulation was directed between AF3 and F3 (left PFC), and between AF4 and F4 (right PFC). There were 150 stimuli delivered to each site. The stimuli consisted of 75 single pulses (unconditioned *test* stimuli) and 75 paired‐pulses (LICI: conditioned stimuli followed by test stimuli). Between these stimuli, there was an ISI of 3 s, and to minimise any order effects, stimuli application was randomly counterbalanced between subjects.

#### 2.2.5 Electroencephalography data processing

EEG recordings were processed offline using the EEGLAB open source toolbox,^58^ in‐house Matlab scripts (The MathWorks, Inc, Natick, MA) and the Brain Connectivity Toolbox.^16^ Data traces were digitally filtered with a linear Finite Impulse Response (FIR) bandpass filter (1–80 Hz) and notch‐filtered at 50 Hz. Continuous EEG data was then segmented into epochs from 50 ms (post‐TMS stimuli) to 1,000 ms. Following epoching, the data was de‐meaned (i.e., the average of the entire epoch was subtracted from each time point) as described by Rogasch et el. (2017) to attenuate the DC offset and optimise the data prior to the independent component analysis (ICA) correction.^59^ Single and paired‐pulse conditions were examined separately. Epochs contaminated by movement artefacts (such as twitching/yawning) were removed by a trained research assistant. To remove eye‐blinks, lateral eye movement and auditory artefacts, we conducted ICA and manually removed up to three of the largest components containing these artefacts (if necessary). On average, across both single and paired conditions, between 1–2 components were removed (only those which contained the previously described artefacts) and the range for component removal was 0–3 components across all participants. Continuous EEG data were then again segmented and epochs from 50 ms (post‐TMS stimuli) to 350 ms were examined via network analysis.

*Measure of LICI:* To obtain the primary measure of LICI for each participant, the area under the curve (AUC) from averaged event‐related potentials (ERP) were quantified. The process is detailed more extensively in our previous publication.^52^ For each subject, the AUC for the time‐frame of 50‐ to 150‐ms post TMS‐pulse, for both single and paired‐pulse conditions were averaged, and compared. EEG inhibition was characterised as the ratio of the AUC of the average paired‐pulse potentials (conditioned) over the average single pulse potentials (unconditioned). The calculation of LICI is reflected by the following equation.

$$\left[1-\frac{\text{Area under rectified curve}\;\left(\text{conditioned}\right)}{\text{Area under rectified curve}\;\left(\text{unconditioned}\right)}\;\right]\times 100$$

### 2.3 Network construction

**FIGURE 2:**
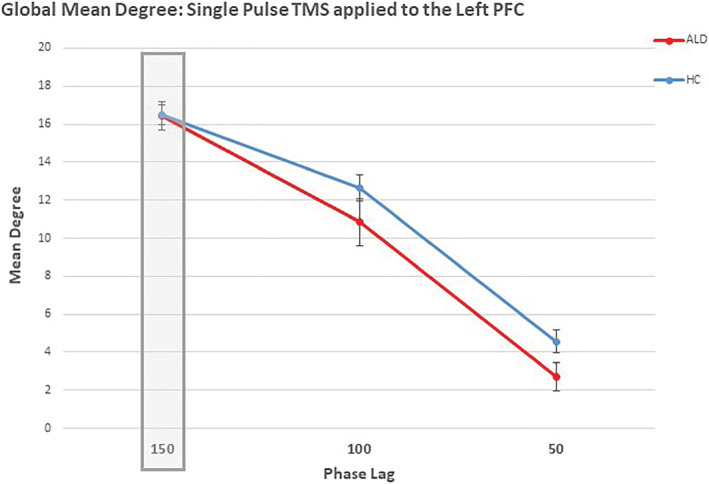
Phase lag: The global mean degree (mean and standard error) across a maximal delay range of 150,100 and 50 ms following single TMS to the left prefrontal cortex (PFC) are presented for Healthy Controls (HC) and individuals with Alcohol dependence in early recovery (ALD). In the current study, the network response to single pulse TMS is considered as a baseline condition and the network response appears to be comparable between groups at a maximal delay of 150 ms

Functional connectivity graphs were generated from the EEG data traces and provide a topographical representation of brain network activity over a fixed time span. The time span consisting of discrete time points, can be defined as all sample points of the original referenced EEG signals *V*_*i*_ *[t] (i = 1*, …, *20)* with *t* between *0* and a given duration *T* (which reflects the time length of the signals). The constructed graph (also called network) consists of a set of vertices (or nodes) *V* and a set of edge weights (or connections) *E* between any two vertices in the graph. The nodes represent signals over the defined time span which are denoted by *V*_*i*_. The edge *e*_*ij*_ between two nodes *V*_*i*_ and node *V*_*j*_, is computed by the normalised pairwise Pearson cross‐correlation coefficient over the given time span for a given time delay *τ*,

$$c\left(\tau \right)=\left(\frac{1}{T-1}\right)\frac{\sum_{t}\left[\left(V_{i}\left[t\right]-{\bar{V}}_{i}\right).\left(V_{j}\left[t-\tau \right]-{\bar{V}}_{j}\right)\right]}{\sqrt{\sum_{t}{\left(V_{i}\left[t\right]-{\bar{V}}_{i}\right)}^{2}}.\sqrt{\sum_{t}{\left(V_{j}\left[t-\tau \right]-{\bar{V}}_{j}\right)}^{2}}}$$

where $\bar{V_{i}}$ is the time average of *V*_*i*_. To account for cross‐cortical conduction times and neurophysiological processes,^60^ the delay *τ* ranged between 0 and 150 ms and was chosen as the delay time maximising the cross correlation. Consideration of this maximal delay *τ* (up to 150 ms) led to the construction of a directed network. Notably, although quite rare, when the pairwise delay approached 150 ms (i.e., between distal electrodes), it was reasonable to extract meaningful connectivity data from 150 ms (when examining the 300‐ms epoch). The global mean degree was examined across a maximal delay range of 150, 100 and 50 ms when single TMS was applied to the left PFC, see Figure 2. In the current study, the single‐pulse condition reflects a baseline condition, as such, based on the data exhibited in Figure 2, the network response appears to be comparable between the groups when a maximal delay lag of 150 ms is applied.

**FIGURE 3:**
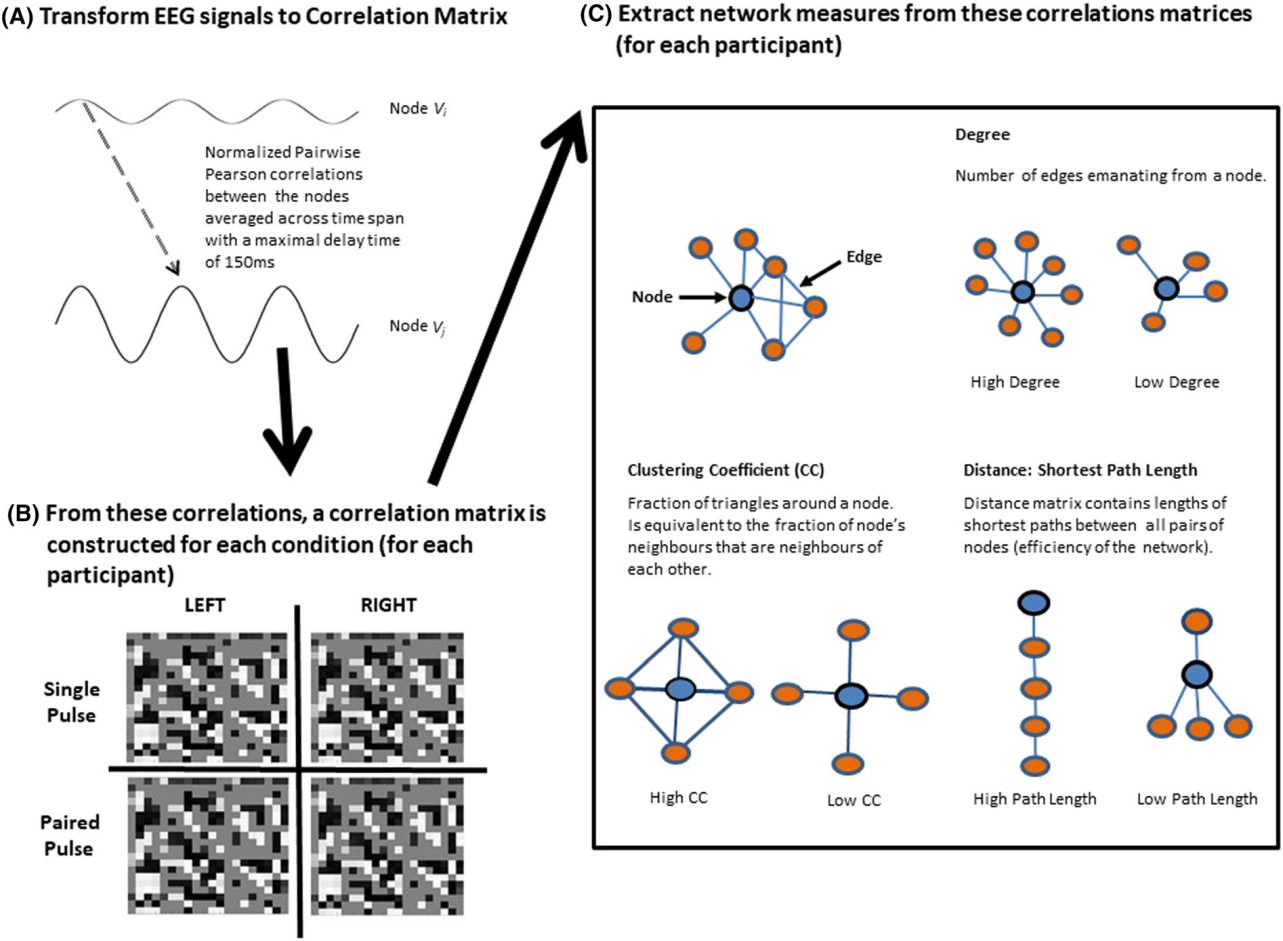
Network analysis pipeline. (A) Electroencephalography (EEG) signals were acquired throughout the transcranial magnetic stimulation (TMS)‐EEG session. Normalised pairwise cross‐correlations between each of the signals were used to construct a correlation matrix. (B) For each participant, individual correlation matrices were constructed for each Stimulation Site (left prefrontal cortex and right prefrontal cortex) and pulse type (single pulse and paired‐pulse). (C) From these correlation matrices, network measures were extracted: mean degree, clustering coefficient, distance and shortest path length (local and global efficiency). These network measures were then used to compare global features of network response to a TMS perturbation across the individuals with alcohol dependence in early recovery (ALD) and healthy controls (HC)

An edge *e*_*ij*_ is then defined as the maximum of *c(τ)* over all *τ*. If *e*_*ij*_ *> e*_*ji*_, only *e*_*ij*_ is kept and *e*_*ji*_ is set to zero. Defining *e*_*ij*_ in this manner represents statistical dependence of the signals between the nodes over the fixed time span. This results in a graph which is both *directed* (i.e. all edges are directed from one node to another) and *weighted* (retaining the edges' correlation co‐efficient index). Individual networks were constructed for each of the participants. Please refer to Figure 3 for an illustration of the network construction.

**FIGURE 4:**
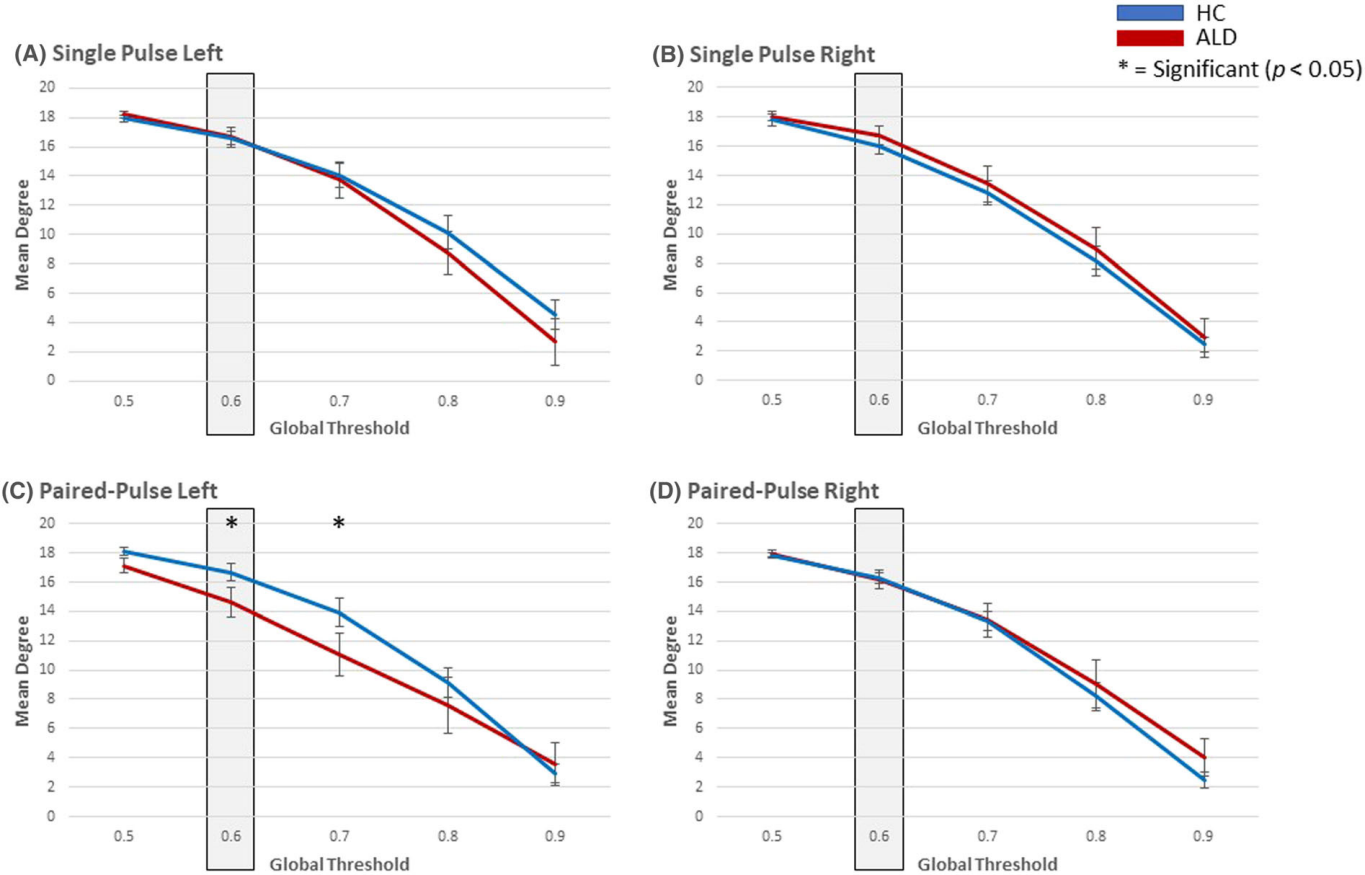
Determining the global threshold: To identify the optimal threshold to apply for network construction, the network response (global mean degree) to the transcranial magnetic stimulation (TMS) perturbation across stimulations site (left and right PFC) and pulse type (single and paired‐pulse) were compared between healthy controls (HC) and individuals with Alcohol dependence in early recovery (ALD) for a range of global thresholds (0.5 through to 0.9). It is notable that significantly altered topological features of the network following paired‐pulse (LICI inhibitory pulse) emerge at a threshold of 0.6 while no difference is observed in the single pulse condition

For each network, a global threshold of 60% was set to remove weak or spurious correlations (i.e., those edges whose weights are close to 0) and thus allow identification of topological properties of the network. A fixed threshold allows an examination of the average number of active connections between nodes and allows us to identify how this differs between the groups. Before settling on the 60% absolute threshold, the global mean degree was examined across a range of thresholds for both stimulation sites (left and right PFC) and both pulse‐type (single and paired‐pulse), see Figure 4. In the current study, the single‐pulse condition is considered the baseline condition, therefore, based on the data shown in Figure 4, we noted the different topological features of the network following the LICI perturbation emerged when the threshold was set at 60% (and similar patterns at 70%). Considering that this is an exploratory study, utilising novel combined TMS‐EEG techniques with network analysis, it is recommended that future studies explore the optimal methods of thresholding for TMS‐EEG studies, as a more extensive examination was not within the scope of the current paper.

#### 2.3.1 Degree (*k*)

In a directed network, the degree *k* of a node denotes the number of edges, or links connecting inward (in‐degree) and outward (out‐degree) from the node. Nodes of high degree demonstrate increased connectivity with the other vertices and may represent hubs in the network.

#### 2.3.2 Clustering coefficient

The clustering coefficient $C_{i}$ quantifies the occurrence of two neighbors of the same node $V_{i}$ being connected, which produces a triangle in the graph. $C_{i}$ presents information regarding local connectivity and structure within a network.^61^ If *t*_*i*_ is the number of triangles that node $V_{i}$ participates in, then $C_{i}$ = 2*t*_*i*_/ $k_{i}$ ( $k_{i}$ − 1), where $k_{i}$ is the degree of node $V_{i}$. Global clustering coefficient computes the average clustering coefficient of all nodes.

#### 2.3.3 Path length, local efficiency and global efficiency (measure of integration)

The characteristic path length *L*_*ij*_ between two nodes refers to the minimum number of edges which is required to pass from node *V*_*i*_ to node *V*_*j*_ (i.e., topological distance) and is also described as the shortest path length. We set $L_{\mathrm{ij}}=\infty$ for any disconnected node pairs *V*_*i*_ and *V*_*j*_. The global efficiency *GE* is defined as the average inverse shortest path length distance in the network $\mathrm{GE}=\frac{1}{\left|V\right|\left(\left|V\right|-1\right)}\sum_{i,j,j\neq i}L_{\mathrm{ij}}^{-1}$. The local efficiency *LE* is a bit more delicate, defined as the average inverse shortest path length of the sub‐graph that includes all the neighbors of a node, but with the node itself removed (otherwise all shortest path lengths would be either 1 or 2). Local efficiency is thus related to the clustering coefficient, for example, if all of a node's neighbors are linked then both its clustering coefficient and its local efficiency are maximal, and equal 1. These efficiency measures reflect the transfer of information and capacity for integrated processing of the network, both globally (for the GE measurement) and locally (for the LE measurement).

### 2.4 Statistical analysis

Comparability of the basic demographics of the ALD group and healthy controls was assessed using independent t‐tests for continuous variables and *χ* ^2^‐tests for categorical variables (Table 1). All data analyses were performed using IBM SPSS Statistics 28 and tests were run at alpha level of 0.05. There were no significant violations of homogeneity of regression or unequal variance. Global network metrics (mean degree, clustering coefficient, local and global efficiency) were extracted for each participant (described in Table 2) and a three‐way mixed measures ANOVA pipeline^62^ guided by the process described in Laerd Statistics (https://statistics.laerd.com) was conducted to identify the presence of significant interactions between group (ALC vs. HC), pulse (single pulse vs. paired‐pulse) and side (left vs. right PFC stimulation).

**TABLE 2:** Mean (unadjusted) and standard deviation of the network response to the TMS stimuli applied to the frontal cortex under the single and paired‐pulse conditions

	Healthy control mean ± SD	Alcohol dependent post‐detox mean ± SD
**Left single pulse**	*n* = 16	*n* = 11
Mean degree	16.58 ± 1.91	16.65 ± 2.34
Mean cluster	0.45 ± 0.04	0.45 ± 0.05
Local efficiency	0.66 ± 0.06	0.67 ± 0.03
Global efficiency	0.68 ± 0.04	0.66 ± 0.03
**Left paired‐pulse**	n = 16	n = 11
Mean degree	16.55 ± 2.46	14.50 ± 3.58
Mean cluster	0.45 ± 0.05	0.41 ± 0.08
Local efficiency	0.66 ± 0.06	0.59 ± 0.11
Global efficiency	0.67 ± 0.06	0.62 ± 0.09
**Right single pulse**	*n* = 13	*n* = 11
Mean degree	16.31 ± 1.76	16.71 ± 2.08
Mean Cluster	0.45 ± 0.03	0.45 ± 0.04
Local efficiency	0.66 ± 0.04	0.67 ± 0.04
Global efficiency	0.66 ± 0.04	0.68 ± 0.04
**Right paired‐pulse**	*n* = 12	*n* = 12
Mean degree	16.26 ± 1.23	16.17 ± 2.16
Mean cluster	0.45 ± 0.02	0.44 ± 0.05
Local efficiency	0.65 ± 0.03	0.65 ± 0.05
Global efficiency	0.64 ± 0.04	0.66 ± 0.04

Across these network metrics, Binomial Logistic Regression and Receiver Operating Characteristic (ROC) Curve analysis were applied to ascertain the likelihood for these network metrics (predictors) to successfully discriminate between participants with ALD or HC. Finally, Pearson's correlation coefficients were examined to assess whether global network metrics correlated with clinical features of alcohol dependence (in the ALD population) according to Total Score on the severity of alcohol dependence questionnaire (SADQ) and measures of cortical inhibition (calculations described in Naim‐Feil et al., 2016). Additionally, to address any potential variability due to the range in duration of abstinence, Pearson's correlation was applied to examine whether there were any significant associations between duration of abstinence and any of the network metrics. No significant correlations were observed, and therefore, it was not necessary to include duration of abstinence in the statistical analysis below as a covariate.

## 3 RESULTS

### 3.1 Transcranial magnetic stimulation‐evoked global network response comparisons between groups (mean and standard error of the group data [ALD: *N* = 10; HC: *N* = 12] included in the three‐way mixed ANOVA are presented in Figure 5)

**FIGURE 5:**
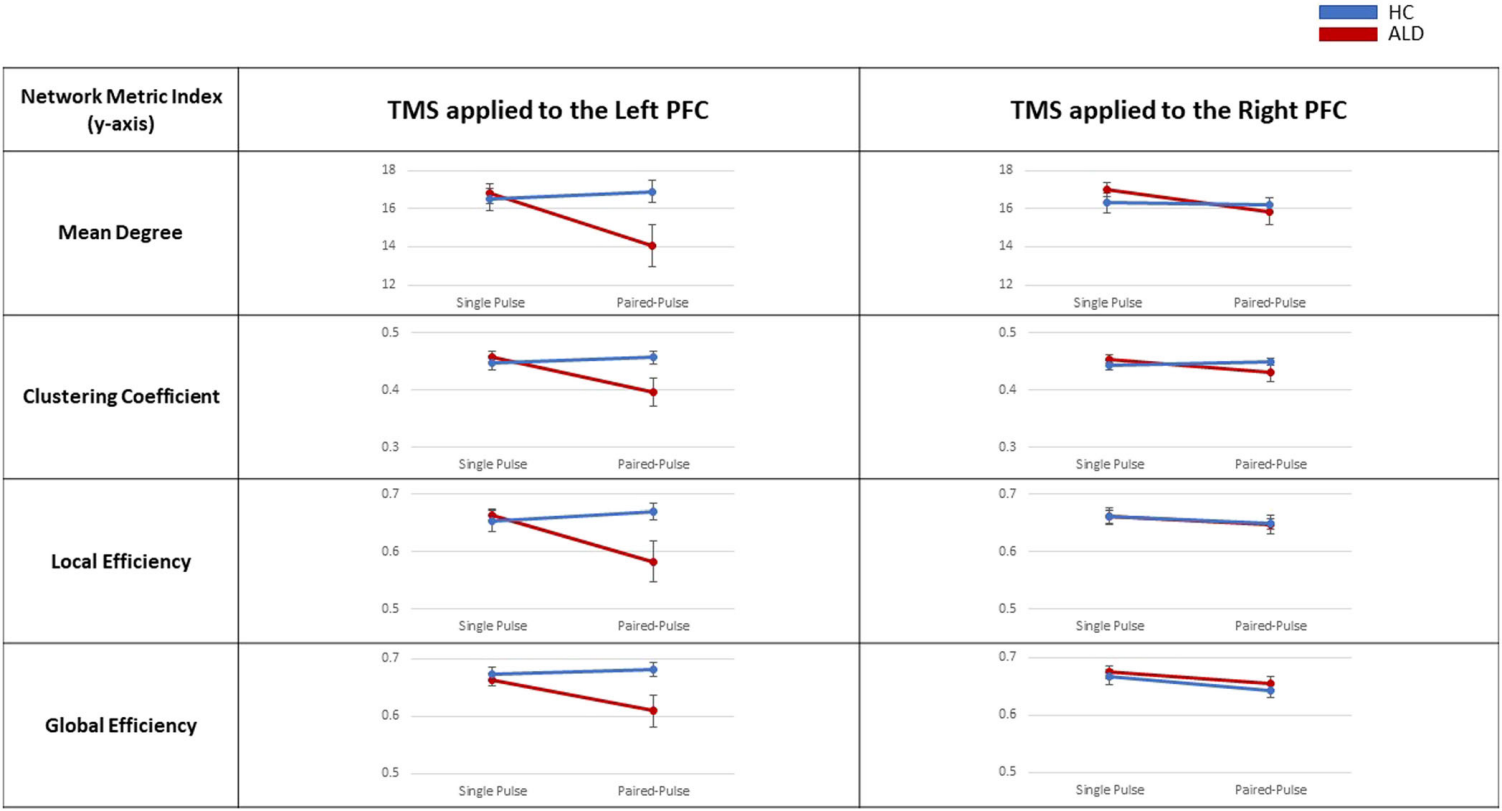
Network response following transcranial magnetic stimulation (TMS)‐perturbations: The unadjusted Mean and Standard Error for all network data included in the 3‐way mixed ANOVA analysis are plotted in this figure. The figure illustrates the network response across the network metrics explored in the current study: Mean degree, clustering coefficient, local efficiency and global efficiency. For each network metrics, the Pulse Type (single and paired‐pulse) have been examined across the stimulation site (left prefrontal cortex (PFC) and right PFC) and compared between the groups (healthy controls (HC) and individuals with alcohol dependence in early recovery (ALD))

#### 3.1.1 Mean degree

There was no statistically significant three‐way interaction observed between Pulse, Side and Group, *F* (1,20) = 1.749, *p* = 0.201, partial eta squared = 0.080. There was a statistically significant two‐way interaction observed between Pulse and Group, *F* (1,20) = 7.971, *p* = 0.01, partial eta squared = 0.285. A nearly statistically significant two‐way interaction was observed between Side and Group, *F* (1,20) = 4.146, *p* = 0.055, partial eta squared = 0.172. All other two‐way interactions were not statistically significant (*p* > 0.05). No significant main effects were observed.

**FIGURE 6:**
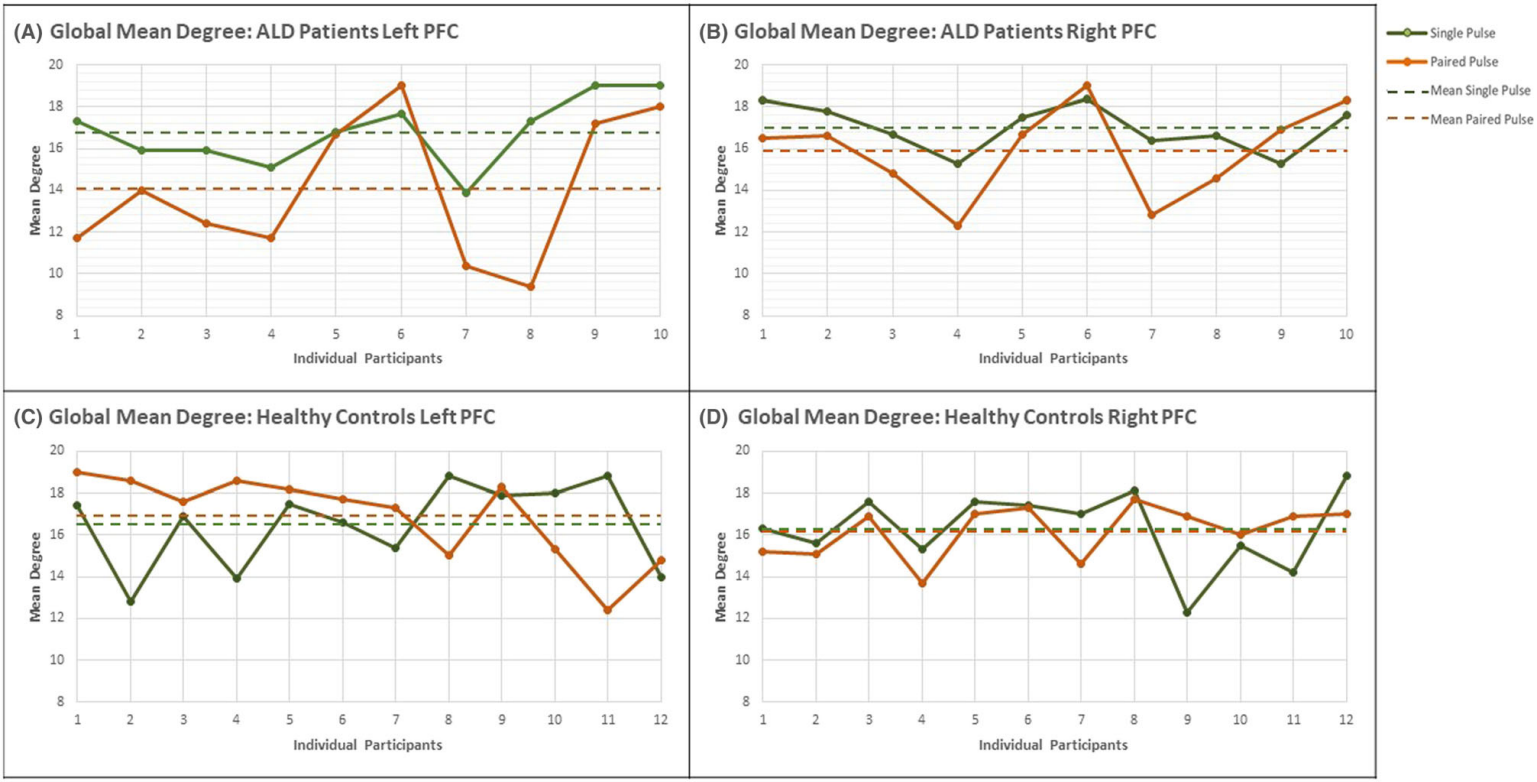
Individual participant network response following transcranial magnetic stimulation (TMS) perturbations: To illustrate the network response on a participant level, the current figure plots all mean degree data included in the three‐way mixed ANOVA analysis. For mean global degree, the pulse type (single and paired‐pulse) have been plotted across each stimulation site (left and right prefrontal cortex (PFC)) for the healthy controls (HC) and individuals with alcohol dependence in early recovery (ALD), separately. Each datapoint corresponds with the individual participant response to the various stimulation parameters. The single pulse response has been plotted along the green line (with the group mean single pulse identified by the broken green line) and the paired‐pulse response plotted along the orange line (with the group mean paired‐pulse identified by the broken orange line). To assist with the visualisation, the continuous data lines were drawn between these discrete datapoints (i.e., the individual participants network response)

As the current study consists of a small sample size, to place context of the averaged data, we also visually inspected the individual participant mean degree network response following the TMS perturbation across the Pulse Types (single and paired‐pulse) for each Stimulation Site (left and right PFC) in Figure 6. Consistent with the grouped averages, there appears to be an altered network response to paired‐pulse applied to the left PFC broadly across the ALD group when compared to the HC (and relative to the single pulse condition).

#### 3.1.2 Clustering coefficient

There was no statistically significant three‐way interaction observed between Pulse, Side and Group, *F* (1,20) = 1.978, *p* = 0.175, partial eta squared = 0.090. A statistically significant two‐way interaction was observed between Pulse and Group, *F* (1,20) = 8.486, *p* = 0.009, partial eta squared = 0.298. All other two‐way interactions were not statistically significant (*p* > 0.05). Statistical significance of a simple main effect was assessed. There was a significant simple main effect of Group following paired‐pulse stimulation, *F* (1,20) = 4.649, *p* = 0.043, partial eta squared = 0.189, but not following single pulse stimulation, *F* (1,20) = 1.261, *p* = 0.275, partial eta squared = 0.059. Pairwise comparisons were performed for these statistically significant simple main effects. Sidak corrections were made with comparisons within each simple main effect. Adjusted *p*‐values, estimated mean (*EM*) and standard error (*SE*) are presented. In response to paired pulse stimulation, the HC presented with an increased index of clustering coefficient (*EM* = 0.453, SE = 0.012) compared to ALC (*EM* = 0.413, *SE* = 0.014), with a mean difference of 0.04, 95% CI [0.001, 0.078], *p* = 0.043. Within the ALC group, the clustering coefficient index in response to the single‐pulse (*EM* = 0.456, *SE* = 0.007) was increased compared to the paired‐pulse (*EM* = 0.413, SE = 0.014), a mean difference of 0.043, 95% CI [0.016, 0.069], *p* = 0.003, while no significant mean difference was identified in the healthy controls.

#### 3.1.3 Local and global efficiency (LE and GE, respectively)

With respect to local efficiency, there was a statistically significant three‐way interaction between Pulse, Side and Group, *F* (1,20) = 5.564, (*p* = 0.029, partial eta squared = 0.218). When compared across groups, there was a statistically significant simple two‐way interaction between Pulse and Side for the alcohol group, *F* (1,9) = 5.200, (*p* = 0.049, partial eta squared = 0.366), but not for healthy controls, *F* (1,11) = 1.036, (*p* = 0.331, partial eta squared = 0.086). When we compared across stimulation sites, there was a statistically significant simple two‐way interaction between Pulse and Group following stimulation to the left PFC, *F* (1,21) = 5.847, (*p* = 0.025, partial eta squared = 0.218), but this was not evident following stimulation to the right PFC, *F* (1,21) = 0.052, (*p* = 0.822, partial eta squared = 0.002). To further explore this relationship, a two‐way ANOVA was applied and paired‐pulse administered to the left PFC was statistically significantly different between groups, *F* (1,25) = 4.533, (*p* = 0.043, partial eta squared = 0.153), but was not significantly different following single‐paired pulse, *F* (1,25) = 0.062, *p* = 0.805, partial eta squared = 0.002. To examine the differences between groups, a simple pairwise comparison was run between groups following paired‐pulse administration to the left PFC. A Sidak adjustment was applied. Local efficiency following paired‐pulse administration to the left PFC for ALC was 0.59 (*SD* = 0.11) and healthy controls was 0.66 (*SD* = 0.061). There was a statistically significant mean difference between local efficiency of 0.071, 95% CI [0.002, 0.140], *p* = 0.043.

With regards to Global Efficiency, there was no statistically significant three‐way interaction observed between Pulse, Side and Group, *F* (1,20) = 2.943, *p* = 0.102, partial eta squared = 0.128. A statistically significant two‐way interaction was observed between Side and Group, *F* (1,20) = 6.359, *p* = 0.02, partial eta squared = 0.241. All other two‐way interactions were not statistically significant (*p* > 0.05). The statistical significance of simple main effects was assessed. There was a significant simple main effect of Group at the left side of stimulation, *F* (1,20) = 6.181, *p* = 0.022, partial eta squared = 0.236, but not at the right side of stimulation, *F* (1,20) = 0.704, *p* = 0.411, partial eta squared = 0.034. Pairwise comparisons were performed for these statistically significant simple main effects. Sidak corrections were made with comparisons within each simple main effect. Adjusted *p*‐values, estimated mean (*EM*) and standard error (*SE*) are presented. In response to left stimulation to the PFC, the HC presented with increased global efficiency (*EM* = 0.68, *SE* = 0.011) compared to ALC (*EM* = 0.64, *SE* = 0.012), a mean difference of 0.04, 95% CI [0.006, 0.074], *p* = 0.022.

### 3.2 Transcranial magnetic stimulation‐evoked global network response to predict group membership

#### 3.2.1 Binomial logistic regression

Following this, binomial logistic regressions were performed to establish the effects of the various stimulation sites (left and right PFC) and pulse type (paired‐pulse and single pulse) across each of the network measures (MD, CC, LE and GE) could predict the likelihood of the participants being in the ALD group. For the binomial logistic regression, the network data were standardised (according to the z‐score: network metric value minus the mean network metric, divided by the standard deviation). For Local Efficiency, the logistic regression model was not statistically significant, *χ* ^2^(2) = 6.047, p < .192. The model explained 32% (Nagelkerke *R* ^2^) of the variance in the ALC group and correctly classified 68% of cases. Sensitivity was 60%, specificity was 75%, positive predictive value was 69% and the negative predictive value was 67%. Local efficiency following TMS applied to the left PFC was statistically significant as an independent predictor (as shown in Table 3). Whereby, each unit reduction in Local Efficiency increases the odds of being in the alcohol‐dependent groups by a factor of 3.759 (based on inverted odds ratio). Therefore, decreasing Local Efficiency was associated with an increased likelihood of membership in the ALD group. As this is an exploratory study, it is notable that following TMS applied to the left PFC, Global Efficiency followed a remarkably similar pattern, but is not considered a significant predictor at *p* = 0.052. MD and CC were also independently assessed via binomial logistic regression and were both found to not contribute significantly to the model.

**TABLE 3:** Logistic regression predicting likelihood of alcohol‐dependence post‐detoxification group membership based on standardised local efficiency (LE) values

	B	SE	Wald	df	p	Odds ratio (inverted)	Odds ratio lower CI (95%)	Odds ratio upper CI (95%)
LE single pulse left	0.191	0.537	0.127	1	0.722	0.83	0.288	2.364
**LE paired‐pulse left**	**−1.323**	**0.661**	**4.005**	**1**	**0.045**	**3.76**	**1.03**	**13.70**
LE single pulse right	−0.326	0.584	0.311	1	0.577	1.39	0.44	4.35
LE paired‐pulse right	0.440	0.577	0.581	1	0.446	0.64	0.21	2.00
Constant	−0.185	0.498	0.138	1	0.710	1.20		

*Note:* The local efficiency odds ratio and the confidence interval (CI) presented are the inverted odds ratio values.

#### 3.2.2 Area under Receiver Operating Characteristic curve

When paired‐pulse stimulation was applied to the left PFC, the area under the ROC curve for 1. Local Efficiency was 0.742 (95% CI, 0.525 to 0.958), 2. Global Efficiency was 0.758 (95% CI, 0.542 to 0.975), 3. Mean Degree was 0.767 (95% CI, 0.552 to 0.981) and 4. Clustering Coefficient was 0.754 (95% CI, 0.536 to 0.972), which is an acceptable discrimination according to Hosmer et al (2013). For single‐pulse stimulation to either stimulation site and paired‐pulse stimulation to the right PFC, the area under the ROC curve for all network measures ranged between 0.39 to 0.59 which is considered poor discrimination.

### 3.3 Transcranial magnetic stimulation‐evoked global network response and clinical features of alcohol dependence

*Site 1. Left PFC*. In the single pulse condition, the total score on the Severity of Alcohol Dependence Questionnaire (SADQ) was negatively related to both MD (*r* = −0.800, *n* = 11, *p* = 0.022) and CC (*r* = −0.702, *n* = 11, *p* = 0.016). Similarly, in the paired‐pulse condition, total score on the Severity of Alcohol Dependence Questionnaire (SADQ) was negatively related to both MD (*r* = −0.620, *n* = 11, *p* = 0.042) and CC (*r* = −0.662, *n* = 11, *p* = 0.027). No significant correlations between any network metrics and cortical inhibition were identified.

*Site 2. Right PFC*. In the paired‐pulse condition only, the total score on the Severity of Alcohol Dependence Questionnaire (SADQ) was negatively related to both MD (r = −0.842, n = 11, p = 0.001) and CC (r = −0.784, n = 11, p = 0.004). No significant correlations between any network metrics and cortical inhibition were observed.

## 4 DISCUSSION

This is the first study to utilise an inhibitory paired‐pulse paradigm (LICI) combined with EEG to identify anomalies in network connectivity in a sample of participants with alcohol dependence during early recovery. We find it significant that following single‐pulse stimulation, no differences in network topology were observed between the groups, while application of LICI to the left PFC elicited an altered network response within the ALD group (relative to controls) that was not seen with right sided stimulation. Based on these findings, we suggest that LICI stimuli transiently elicited an ‘inhibitory’ response within the addiction circuitry, while EEG characterised the altered global network response.

Similar to previous fMRI and DTI studies, the current study observed alterations in both global and local integration within locally specialised connectivity (mean clustering coefficient) and in the hub‐like structure (mean degree). Following paired‐pulse stimulation to the left PFC, a decrease in local integration (local efficiency) was found to significantly increase the odds of membership in the ALD group, while network metric classifiers (MD, CC, LE and GE), were capable of adequately discriminating between the groups. Furthermore, in the ALD group, reductions in both locally specialised connectivity (mean clustering coefficient) and hub‐like structure (mean degree) were associated with increased severity of alcohol dependence. When combined, our results support the notion that directly targeting the addiction circuitry (via the LICI perturbation) while utilising EEG technology can provide further insight into clinically relevant altered network topology associated with long‐term alcohol use during early recovery.

### 4.1 Altered network metrics and alcohol dependence

The first network feature explored in the current study was Degree Centrality (hub‐like structure) which represents the integration and processing of network information and communication within the network.^63^ From a clinical perspective, it has been found that a global decrease in nodal degree is associated with longer duration^19^ and with greater severity of alcohol dependence.^34^ In the current study, the ALD group exhibited altered degree centrality in response to the LICI perturbation compared to HC. Moreover, following paired‐pulse stimulation to the left PFC, degree centrality as network classifier could adequately discriminate between the groups. This classification was not observed following single‐pulse or right‐side stimulation. Additionally, in the ALD group, reductions in degree centrality were associated with an increase in alcohol dependence severity. Previous research has demonstrated that short term inebriation elicited increased degree centrality of various nodes in the resting‐state condition,^34^ while long‐term alcohol use was associated with the presence of abnormal functional hubs (varied across regions within the network).^29^ However, emerging neuroimaging studies (fMRI, DTI and EEG) into long‐term alcohol use are still mixed regarding the direction of these network anomalies, with some studies reporting decreased nodal degree in various brain regions^29^ and other studies reporting increased nodal degree.^30^

Another key topological feature of the brain network that has been examined by addiction studies is locally specialised connectivity of the network. The clustering coefficient, which indexes the number of connections existing between a node's nearest neighbours, provides a measure of locally specialised connectivity.^14^ Complex networks present with high clustering, while reduced clustering may indicate a shift towards random organisation. Reduced clustering has been previously associated with increased severity of alcohol use and duration of alcohol dependence.^19^ In the current study, the ALD group presented with a lower clustering coefficient across the network in response to the LICI perturbation but not in response to single pulse stimulation. Following paired‐pulse stimulation (only) to the left PFC, clustering coefficient network metrics were capable of discriminating between the groups. Additionally, in the ALD group, a decrease in clustering coefficient was associated with an increase in alcohol dependence severity. Currently, there are only limited fMRI and DTI findings regarding group differences in clustering coefficient following alcohol exposure. Rank‐ordered differences were found with values of nodal clustering being lowest in the ALD group (compared to healthy controls and unaffected siblings of the ALD participants).^25^ Reduced clustering has also been observed in an abstinent addicted population^20^ and in individuals with familial risk of developing alcohol dependence.^23^ However, a number of other studies failed to observe any significant group differences on global measures of clustering.^19^, ^30^

With regard to the preliminary EEG studies, data modelling was applied to EEG data to ascertain the predictive value of detecting an ALD group compared to healthy group based on network features. Reduced clustering coefficient was found to reliably predict the ALD group under various stimulus conditions.^64^ This was supported by one EEG study in males with alcohol dependence after approximately 28 days of detoxification, which identified reduced clustering (in the low beta band),^36^ while the second EEG‐MEG study observed group difference in local connectivity (decreased clustering at the posterior sites and increased clustering at the frontal sites) but no significant differences in global connectivity.^35^ These studies, when combined with the current study, provide initial evidence of compromised locally specialised connectivity associated with long‐term alcohol use. Additional studies are required to further characterise how these anomalies relate to relapse and recovery.

A prominent focus of addiction network studies is the efficiency (a measure of information transfer) of the brain network. Measure of Efficiency, which is represented by a network with short characteristic path length, provides a measure of parallel information transfer between nodes and integrated processing.^14^ High level functioning relies on efficient information transfer, which is also beneficial for cognitive control and executive function.^18^, ^65^ From a clinical perspective, decreased global efficiency has been found to relate to a longer history of alcohol dependence,^19^ while increased local efficiency was related to greater duration of abstinence.^28^ In the current study, participants with ALD presented with reduced local efficiency in response to the LICI perturbation but not in response to single pulse stimulation. Moreover, it was found that as local efficiency reduced, the predictive odds of being in the ALD group increased. With regards to global efficiency, participants with ALD demonstrated reduced global efficiency in response to left sided stimulation. Following paired‐pulse stimulation to the left PFC (only), local and global efficiency network classifiers were able to adequately discriminate between the groups. This is consistent with fMRI and DTI studies which have generally identified that decreased efficiency is associated with chronic alcohol use^18^, ^28^ and persists during abstinence,^20^ while increased efficiency was found to be related to remission.^22^ Additionally, individuals considered at risk of developing alcohol dependence (familial risk and children with foetal alcohol spectrum disorder) also presented with significantly reduced efficiency.^23^, ^24^ However, these significant group differences were not identified by all studies.^19^, ^30^

While many fMRI and DTI studies predominantly identify reduced global efficiency, only a small number of EEG studies have characterised these network anomalies and present mixed findings. In terms of short‐term alcohol consumption, a preliminary resting‐state EEG study examined network connectivity in social drinkers following alcohol administration as compared to placebo in healthy social drinkers.^34^ Short‐term inebriation resulted in increased global efficiency in the resting‐state network.^34^ With regards to long‐term alcohol exposure, two studies examined network connectivity of males with alcohol dependence after approximately 28 days of detoxification.^35^, ^36^ The first EEG study examined network response during a working memory task and identified shorter characteristic path length and increased global efficiency.^36^ The second study utilised EEG‐MEG during resting‐state and observed no group differences in global efficiency. However, differences in local efficiency were identified, specifically, the presence of decreased efficiency at the posterior sites and increased efficiency in the frontal sites of the ALD group.^35^ Therefore, while fMRI and DTI neuroimaging studies provide compelling evidence of reduced local and global efficiency associated with long‐term alcohol use, the findings from EEG studies are less consistent. In the current examination of local efficiency, there were no significant group differences observed in response to the single stimuli; the ALD group exhibited reductions in local efficiency, which are comparable with the fMRI and DTI studies, only when the paired‐pulse inhibitory paradigm was applied to the addiction network. Notably, while the findings regarding global efficiency followed a similar pattern (but did not reach significance regarding the comparison between LICI and single pulse administration), there were significant differences in network response to the TMS perturbation between groups, it is anticipated that a future study with a larger sample size is required to further explore the response to LICI (as compared to the single pulse).

Therefore, the current study identified a shift in the balance of network integration and segregation following a paired‐pulse TMS perturbation to the left PFC. Human brain networks have been found to exhibit small world networks which rely on an efficient balance between information segregation and integration (via high clustering and high global efficiency).^66^ Across a range of psychiatric disorders, a loss to small‐world organisation have been identified, with shifts in small‐world features depending on the pathology of the disorder.^67^ In the current study, we have identified that ALD patients exhibit altered small‐world organisation (reductions in both efficient segregation and integration), which persist beyond detoxification. Therefore, we suggest that high‐cost elements of brain network organisation (such as high clustering and high efficiency)^68^ appear to be compromised in the ALD patient group, and moreover, the persistence of this altered topological organisation (beyond detoxification) may indicate an enduring risk to the economic organisation of the brain network.

### 4.2 Altered brain networks, brain stimulation paradigms and models of addiction

Recent studies have provided preliminary evidence for the utility of combined neuroimaging‐brain stimulation techniques to map and explore network change.^48^, ^69^ The present study is the first to apply LICI to examine global network anomalies underlying alcohol dependence. According to neurobiological models, acute alcohol exposure facilitates GABAergic inhibitory activity resulting in an overall inhibitory effect.^70^, ^71^, ^72^, ^73^ Chronic alcohol exposure elicits a compensatory response, whereby the brain seeks to restore equilibrium in neural function, which leads to neuroadaptation in the mesocorticolimbic ‘addiction’ circuitry, and subsequent suppression of GABAergic neurotransmission and facilitation of glutamatergic neurotransmission.^13^, ^74^, ^75^ Cortical inhibition (CI), the neurophysiological process wherein GABA inhibitory interneurons selectively dampen activity of other neurons in the cortex, is critical to understanding the development of alcohol dependence.^76^ It has been found that application of LICI, a paired‐pulse inhibitory TMS paradigm, is capable of non‐invasively characterising CI of the prefrontal cortex.^41^, ^42^, ^77^ This approach has been utilised to identify altered cortical excitability within the frontal cortex of participants with alcohol dependence post‐detoxification.^52^ However, as the frontal cortex is intricately interconnected with widely distributed brain regions, it was important to explore whether the compensatory response (i.e. the neurophysiology of widespread suppression of GABAergic neurotransmission) may also be identified at the network level.

Recent studies have examined whether the neurophysiology of short‐term alcohol exposure can be reflected by alterations in global connectivity. One preclinical study identified alterations in network organisation following acute ethanol exposure.^78^ This was also observed in a study of social drinkers, whereby acute ethanol had a measurable effect on brain networks and appears to increase network density (hub‐like behaviour) and global efficiency (global integration).^34^ Our results indicate that the inhibitory network surrounding the left FPC is considerably weakened (reduced MD, CC, LE and GE) in the ALD group in early recovery, when compared to the inhibitory network of both the left and right PFC in the HC group. Moreover, an increase in global connectivity has been identified as a potential indicator of remission in those with long‐term abstinence^22^ and reduced the likelihood of relapse.^27^ This suggests a striking similarity with the allostasis model of alcohol dependence,^79^ albeit from a network perspective. Similar to the allostasis model, these studies may indicate the presence of a compensatory network response (to maintain stability of the network) following repeated alcohol exposure, resulting in network‐adaptations which may persist in early recovery. However, notably, we did not observe a direct relationship between cortical inhibition and network features. Based on this, we suggest that quantification of the network architecture provides insight into the communication structure that coordinates distributed neural processes within the addiction network; this differs from the measure of cortical inhibition, which is a localised neurophysiological response within a specific region of the network. Thus, we propose that network science applied to TMS‐EEG could contribute meaningfully to current allostasis models of alcohol dependence by quantifying how the pathways of communication between altered brain regions may be compromised following repeated alcohol use.

The efficacy of brain stimulation techniques (such as repetitive TMS and transcranial direct current stimulation) to alter network topology^80^, ^81^, ^82^, ^83^ has also been recently explored. Specifically, it was found that brain stimulation (both transcranial direct current stimulation and theta burst stimulation) was capable of altering global network connectivity features, such as global efficiency, clustering coefficient and strength of the network.^27^, ^80^, ^81^, ^82^ Therefore, we propose that in addition to TMS‐EEG being a potentially useful experimental tool for identifying network biomarkers, there are potential treatment possibilities in administering brain stimulation techniques (such as tDCS and TMS) as a therapeutic tool for altering network properties and potentially reducing clinical symptoms of alcohol dependence.^84^, ^85^ Further research is required to elucidate the role of brain stimulation in understanding the network features as well as identifying the potential therapeutic efficacy of utilising brain stimulation to treat addiction.

### 4.3 Study limitations

Although these findings provide preliminary EEG evidence of the presence of altered network connectivity among individuals with alcohol dependence in early recovery, various methodological issues should be addressed. First, in applying graph theory to neuroimaging data there are numerous approaches to network construction, reference choice and thresholding considerations. The current study utilised a widely applied network construction technique (Pearson correlation) in which cross‐correlation data are extrapolated from EEG traces^86^ and set a global threshold of 60% to remove spurious correlations. In response to the single pulse, the network mean degree did not significantly differ between groups. Therefore, we suggest that the single‐pulse networks were comparable between the groups and could be considered the baseline condition. As such, the described network construction is appropriate for comparing the response to the paired‐pulse condition and assessing how this inhibitory TMS perturbation altered the network. Second, with regards to the TMS‐EEG technique, whilst TMS‐EEG induced inhibition is proposed to be cortical in nature, it is important to note that other sources, such as the auditory stimuli (from the click associated with the pulse) may also stimulate the auditory cortex and associated regions. However, previous TMS‐EEG studies have observed that white noise at 96 dB (which we employed) should be sufficient to eliminate these effects.^41^ Third, in the current study, consistent with a previous study, there appears to be unilateral lateralization in the elicited network response which was prominent in the left hemisphere.^29^ There is still not extensive research into laterality associated with risk factors for addictive behaviours^87^; however, past brain stimulation studies have demonstrated that administration of TMS pulses applied to the left hemisphere was capable of reducing levels of craving across various addictive behaviours (as reviewed in Feil and Zangen, 2013). Therefore, given that the current study is preliminary in nature, with a small sample size, we propose that future studies expand on these findings and further explore the potential laterality effects of TMS to the left and right hemispheres in examining the global networks associated with alcohol dependence post‐detoxification. In addition, in this preliminary study, abstinence was ascertained by over‐the‐phone screening by a trained research assistant, and then, as a secondary screening measure, the Timeline Follow Back calendar was administered at the testing session to confirm abstinence. However, we suggest that future larger scale studies consider including a measure of ethyl glucuronide as a physiological measure of abstinence. Furthermore, as per the nature of cross‐sectional addiction studies, it is very difficult to assert whether the identified altered network connectivity is a direct result of chronic alcohol exposure, or due to pre‐existing vulnerabilities, or quite possibly, the combination of both. We anticipate that future longitudinal studies could expand on these findings and examine whether altered brain networks are a possible predictor of subsequent vulnerability to alcohol dependence, or whether it occurs predominantly as a consequence of long‐term alcohol exposure. Regardless, results from the current study are promising and provide initial proof of concept for utilising TMS‐EEG paradigms to examine anomalies in brain connectivity associated with alcohol dependence; however, these findings are preliminary and further studies are required to confirm these findings.

### 4.4 Conclusion

The current study is the first to directly target the addiction circuitry (via an inhibitory TMS paradigm) whilst simultaneously utilising EEG to quantify anomalies in network topology associated with persisting features of alcohol dependence. Identified network anomalies included reduced global integration, locally specialised connectivity, and hub‐like structures. These network alterations were related to clinical features of alcohol dependence, whereby reduced global integration was predictive of high alcohol use, while decreased locally specialised connectivity and hub‐like structures were associated with increased alcohol use severity. Therefore, whilst preliminary, the current study provides compelling evidence of the potential efficacy of TMS‐EEG combined with network science to identify network biomarkers associated with alcohol dependence and early recovery. Further studies are required to confirm and extend these preliminary findings. A better understanding of the global features of the network may contribute to current neurobiological models of alcohol dependence. Additionally, it is anticipated that these network biomarkers may be used to identify risk factors associated with the development of alcohol dependence and as potential markers of treatment efficacy.

## AUTHOR CONTRIBUTIONS

**Jodie Naim‐Feil:** Conceptualization, Methodology, Formal Analysis, Writing–Original Draft**. Paul B. Fitzgerald:** Conceptualization, Methodology, Writing–Review and Editing, Supervision, Project administration. **Mica Rubinson**: Formal Analysis, Writing–Review and Editing. **Dan I. Lubman:** Project administration, Writing–Review and Editing. **Dianne M. Sheppard:** Conceptualization, Methodology, Writing–Review and Editing, Supervision. **John L. Bradshaw**: Conceptualization, Methodology, Writing–Review and Editing, Supervision. **Nava Levit‐Binnun**: Formal Analysis. **Elisha Moses**: Conceptualization, Methodology, Writing–Review and Editing, Formal Analysis, Supervision.

## CONFLICT OF INTEREST

Dr. Jodie Naim‐Feil was a recipient of the Senior Post‐doctoral Fellowship at the Weizmann Institute, the Curwen‐Lowy Post‐doctoral Fellowship and the Clore Post‐doctoral Fellowship that supported the further analysis conducted for the study. Prof. Paul B. Fitzgerald has received equipment for research from MagVenture A/S, Nexstim, Neuronetics and Brainsway Ltd and funding for research from Neuronetics. He is a founder of TMS Clinics Australia. The study was supported by Dr. Nava Levit‐Binnun's Israel Science Foundation grant (No. 1169/11) and by the National Institute of Psychobiology in Israel. Prof. Elisha Moses is supported by the Minerva Foundation (Germany) and by the Israel Science Foundation. There is no conflict of interest to declare. All of the sponsors who donated to the authors involved in the study had no role in study design; in the collection, analysis and interpretation of data; in the writing of the report; and in the decision to submit the paper for publication.

## Data Availability

The data that support the findings of this study are available on request from the corresponding author. The data are not publicly available due to privacy or ethical restrictions.
